# Antioxidant Activity and Dose-Dependent Toxicity of a Traditionally Consumed *Ipomoea pes-caprae* Infusion Evaluated in a Triple-Negative Breast Cancer Xenograft Model

**DOI:** 10.3390/nu18142248

**Published:** 2026-07-09

**Authors:** Karla I. Llerenas-Aguirre, Gustavo A. Hernández-Fuentes, José A. Toscano-Velázquez, Ariana Cabrera-Licona, Fabian Rojas-Larios, Osiris G. Delgado-Enciso, Idalia Garza-Veloz, Héctor R. Galván-Salazar, Carmen Meza-Robles, Mario Ramírez-Flores, Karla B. Carrazco-Peña, José Guzmán-Esquivel, Janet Diaz-Martinez, Margarita L. Martinez-Fierro, Iván Delgado-Enciso

**Affiliations:** 1 Department of Molecular Medicine, School of Medicine, University of Colima, Colima 28040, Mexico; itatti.k@gmail.com (K.I.L.-A.); ghfuentes@ucol.mx (G.A.H.-F.); jtoscano1996@gmail.com (J.A.T.-V.); arianacabrera267@gmail.com (A.C.-L.); frojas@ucol.mx (F.R.-L.); 1933osiris@gmail.com (O.G.D.-E.); mario_ramirez@ucol.mx (M.R.-F.); dra_carrazco@ucol.mx (K.B.C.-P.); 2 Faculty of Chemical Sciences, University of Colima, Coquimatlan 28400, Mexico; 3 State Cancerology Institute of Colima, Health Services of the Mexican Social Security Institute for Welfare (IMSS-BIENESTAR), Colima 28085, Mexico; hector_rgs@hotmail.com (H.R.G.-S.); carmen.qfb@gmail.com (C.M.-R.); 4 Molecular Medicine Laboratory, Academic Unit of Human Medicine and Health Sciences, Autonomous University of Zacatecas, Zacatecas 98160, Mexico; idaliagv@uaz.edu.mx; 5 Clinical Epidemiology Research Unit, Mexican Institute of Social Security, Villa de Alvarez, Colima 28984, Mexico; jose.esquivel@imss.gob.mx; 6 Research Center in Minority Institutions, Florida International University (FIU-RCMI), Miami, FL 33199, USA; jdimarti@fiu.edu; 7 Department of Dietetics and Nutrition, Robert Stempel College of Public Health and Social Work, Florida International University, Miami, FL 33199, USA

**Keywords:** *Ipomoea pes-caprae*, bioactive compounds, phytochemicals, antioxidant activity, triple-negative breast cancer, toxicity

## Abstract

**Background/Objectives:** Triple-negative breast cancer (TNBC) is one of the most aggressive breast cancer subtypes and remains associated with limited therapeutic options and high systemic toxicity from conventional chemotherapy. *Ipomoea pes-caprae* is a coastal medicinal plant traditionally consumed in Mexico for inflammatory and renal disorders and contains bioactive metabolites with reported antioxidant and pharmacological properties. However, its antitumoral activity and systemic safety profile remain poorly understood. This study aimed to characterize the phytochemical composition, antioxidant capacity, antitumoral activity, and toxicity of a traditionally prepared aqueous infusion of *I. pes-caprae* leaves (IPCAE). **Methods:** IPCAE was characterized using phytochemical screening and complementary instrumental analyses. Antioxidant activity was evaluated using the DPPH assay. A randomized preclinical study was performed in mice bearing MDA-MB-231 xenografts treated with IPCAE, cisplatin, or saline control. **Results:** The infusion showed measurable antioxidant activity (72.25 ± 1.25% DPPH inhibition at 1 mg/mL) and a total polyphenol content of 7.29 µg/mg gallic acid equivalents. Phytochemical screening revealed abundant flavonoids and reducing sugars, with moderate saponin content. In vivo, IPCAE produced only a transient and non-significant trend toward slower tumor progression compared with control (*p* = 0.214) and cisplatin (*p* = 0.377). However, marked systemic toxicity was observed, including severe thoracic dermal lesions in 40% of animals and 70% mortality by day 15. Survival was significantly reduced compared with control and cisplatin groups (*p* < 0.001). **Conclusions:** Although IPCAE exhibited antioxidant activity, no statistically significant antitumoral effect was observed under the evaluated conditions. Furthermore, repeated oral administration resulted in marked systemic toxicity, characterized by visible dermal lesions, clinical deterioration, and increased mortality. Therefore, the present findings do not support the use of the evaluated crude preparation as an anticancer intervention. Future studies should focus on detailed toxicological characterization, bioassay-guided fractionation, dose optimization, and identification of the individual metabolites responsible for the observed biological effects. The antioxidant activity demonstrated in this study should be interpreted independently from antitumoral activity, as no causal relationship between these findings was established.

## 1. Introduction

Breast cancer remains the most frequently diagnosed malignancy worldwide with approximately 2.3 million new cases and 670,000 deaths reported in 2022 [1,2]. Among its molecular subtypes, triple-negative breast cancer (TNBC) is one of the most aggressive forms, characterized by the absence of estrogen receptor (ER), progesterone receptor (PR), and human epidermal growth factor receptor 2 (HER2) expression. TNBC accounts for approximately 10–20% of breast cancer cases and is associated with high invasiveness, poor prognosis, rapid progression, and limited therapeutic options [3,4,5]. Although chemotherapeutic agents such as cisplatin remain part of current treatment strategies, their clinical use is frequently restricted by systemic toxicity, resistance, and adverse effects, particularly in advanced disease stages [6,7]. Consequently, the identification of novel bioactive compounds from natural sources has become an important area of oncological and pharmacological research.

Medicinal plants have historically represented a major source of therapeutic agents; however, their biological effects are often assumed to be inherently safe due to their traditional use [8,9]. In recent years, increasing attention has been directed toward validating ethnomedicinal species through experimental pharmacology and toxicology studies, particularly those traditionally used for inflammatory and chronic diseases. Among these species, *Ipomoea pes-caprae* (L.) R. Br., commonly known in Mexico as “riñonina,” “bejuco de playa,” or “campanilla de playa,” has attracted attention because of its extensive use in coastal traditional medicine [10,11].

*I. pes-caprae*, a member of the Convolvulaceae family, is widely distributed along tropical and subtropical coastal regions. In traditional medicine in Colima, Mexico, aqueous infusions prepared from its leaves are commonly used for kidney inflammation or pain, urinary disorders, rheumatism, lumbalgia, fever, dysentery, and asthma. Additionally, topical applications are employed for wound healing, jellyfish stings, and inflammatory conditions [10,12,13]. These traditional uses have motivated increasing scientific interest in evaluating the pharmacological properties and safety profile of this species [10,13].

Several in vitro studies have reported antioxidant, anti-inflammatory, cytoprotective, and wound-healing activities associated with extracts of *I. pes-caprae*, effects that have been attributed primarily to its flavonoid- and phenolic-rich composition [10,12,14]. In contrast, in vivo investigations remain comparatively limited and have focused mainly on antinociceptive, anti-inflammatory, cardiovascular, and reproductive effects in animal models. Some studies have also reported reproductive toxicity and other systemic biological responses following administration of concentrated extracts, suggesting that the plant contains pharmacologically active constituents capable of producing both beneficial and adverse effects depending on the preparation and exposure conditions [10,12,13].

Previous pharmacological studies have demonstrated that extracts and essential oils from *I. pes-caprae* possess diverse biological activities, including antispasmodic, anti-inflammatory, antioxidant, antinociceptive, and wound-healing effects. Certain hydroalcoholic extracts have also shown cardiovascular activity and reproductive toxicity in animal models, suggesting that the plant contains biologically potent metabolites capable of exerting systemic effects [14,15,16]. However, despite these reported biological properties, evidence regarding the potential anticancer activity of *I. pes-caprae* remains limited and fragmented. Most available studies have focused on antioxidant and anti-inflammatory effects, whereas its potential antitumoral activity and systemic safety under experimental conditions have been insufficiently investigated. To our knowledge, no studies have comprehensively evaluated both the antitumoral activity and toxicity of a traditionally prepared aqueous infusion of *I. pes-caprae* in an in vivo TNBC model.

Phytochemical analyses have identified flavonoids such as hyperoside, isoquercetin, and quercetin derivatives, as well as phenolic acids, coumarins, terpenoids, quinones, and saponin-related compounds. Many of these metabolites are recognized for their antioxidant and cytotoxic properties and have been associated with modulation of oxidative stress and inflammatory pathways [17,18]. Because oxidative stress and chronic inflammation are closely linked to carcinogenesis and tumor progression, these phytochemical characteristics provide a rationale for exploring the potential antitumoral effects of this species. Nevertheless, direct experimental evidence supporting such activity remains scarce.

Despite its broad traditional use and pharmacological potential, information regarding the antitumoral activity and systemic safety of *I. pes-caprae* preparations remains limited [15]. Importantly, traditional use does not necessarily guarantee safety under experimental or concentrated conditions, as the biological effects of medicinal plants may vary substantially according to extraction method, concentration, preparation, and administered dose. Accordingly, both efficacy and safety should be systematically evaluated before considering medicinal plants for therapeutic development. This distinction is particularly relevant when evaluating crude extracts for systemic anticancer applications. Therefore, the aim of the present study was to evaluate both the potential antitumoral activity and the systemic safety profile of a traditionally prepared aqueous infusion of *I. pes-caprae* leaves in a TNBC model using MDA-MB-231 cells. By integrating phytochemical characterization with in vivo evaluation, this study sought to provide preclinical evidence regarding both the biological activity and safety of this traditionally consumed medicinal plant.

## 2. Materials and Methods

### 2.1. Preparation of Ipomoea pes-caprae Infusion

Leaves of *Ipomoea pes-caprae* (L.) R. Br. were collected in May 2023 from a coastal area in the municipality of Tecomán, Colima, Mexico (18°54′38″ N, 103°52′26″ W/18.910604° N, 103.873789° W). The region is characterized by a warm sub-humid climate with seasonal rainfall and sandy soils typical of littoral ecosystems. The collection site was located away from urban and industrial zones to minimize the risk of environmental contamination. *Ipomoea pes-caprae* specimens were collected from naturally growing wild coastal populations and were not subjected to any agronomic management prior to harvesting. No cultivation practices, fertilization programs, irrigation systems, or phytosanitary products were applied before collection.

Plant identification was confirmed by comparison with herbarium specimens deposited at the National Herbarium of Mexico (MEXU:311512) [19,20], ensuring taxonomic accuracy. As *I. pes-caprae* is not listed under any protected category in Mexican environmental regulations, no special collection permits were required. The harvested leaves were thoroughly washed with potable water, air-dried for two weeks, and subsequently dehydrated at 40 °C for 32 h in a Novatech^®^ incubator. The dried material was then ground into a fine powder using an industrial blender.

An aqueous infusion was selected because it represents the customary form in which *I. pes-caprae* is prepared and consumed in the coastal region of Tecomán, Colima. During field collection, ethnobotanical information was documented from a local herbal practitioner with more than 40 years of experience in the traditional use of this species, who reported that the infusion is commonly prepared using approximately 12–14 dried leaves in 1 L of water, corresponding to 2.6–2.9 g of dried leaves. This ethnobotanical information was used solely to estimate the amount of plant material evaluated under controlled experimental conditions and to guide selection of the experimental dose [21,22,23]. Using this customary preparation as the reference and considering a standard adult body weight of 70 kg, the estimated exposure corresponded to 37.1–41.4 mg of dried leaves/kg/day. Standard body surface area (allometric) dose conversion (Km human = 37; Km mouse = 3) [24,25] yielded an equivalent murine dose of approximately 457–511 mg/kg/day; therefore, an experimental dose of 500 mg of dried leaves/kg/day was selected.

For infusion preparation, powdered leaves were suspended in distilled water at a ratio of 1:10 (*w*/*v*) and heated to 90 °C for 10 min, following the customary preparation method documented during fieldwork. The mixture was cooled to room temperature, filtered through sterile gauze, and subsequently sterilized using a 0.20 µm nylon Acrodisc^®^ filter (Pall Corporation, Port Washington, NY, USA) under a Class II A2 laminar flow hood.

To preserve the native chemical composition of the plant metabolites, the infusion was freshly prepared immediately before each administration and was not stored, thereby minimizing degradation of thermolabile and oxidizable compounds [26].

For the in vivo experiments, animals received the freshly prepared aqueous infusion adjusted to deliver 500 mg of dried leaves/kg/day (12.5 mg of dried leaves in 0.17 mL for a 25 g mouse). The administered material corresponded to the infusion itself rather than a purified or lyophilized extract. Based on the extraction yield (11.2%), this dose was equivalent to approximately 56 mg/kg/day of lyophilized solids. A lyophilized preparation obtained from the same infusion was used exclusively for phytochemical characterization, chromatographic and spectroscopic analyses.

### 2.2. Preliminary Phytochemical Screening, Thin-Layer Chromatography (TLC), and Spectroscopic Profile

A preliminary phytochemical screening was performed to identify the major classes of secondary metabolites present in the aqueous infusion of *Ipomoea pes-caprae* leaves (IPCAE), following the procedure described by Oloya et al. [27,28], with slight modifications.

The infusion was prepared fresh at the time of administration, following the traditional method to preserve the native phytochemical composition and prevent chemical alterations of the plant material [26]. For analytical purposes, 100 mL of this infusion were prepared according to the traditional procedure and subsequently freeze-dried using a Freeze Dryer IlShinBioBase model TFD8503 (South Korea) to obtain a stable dry extract for further testing.

All qualitative phytochemical tests were conducted using a 0.1 mg/mL methanolic stock solution of the lyophilized extract. The presence of alkaloids was evaluated using Dragendorff’s, Mayer’s, and Wagner’s reagents, which produced characteristic precipitates indicating positive reactions. Flavonoids were detected by the Shinoda test (reaction with concentrated HCl and magnesium) and confirmed by the Marini Bettolo reaction using antimony pentachloride (SbCl_5_) in CCl_4_. Saponins were identified through foam formation and hemolysis tests on 7% blood agar. Tannins were revealed by the appearance of a blue–black coloration upon addition of saturated ferric chloride solution. The presence of terpenoids was confirmed through the Salkowski test, where the addition of concentrated H_2_SO_4_ to the chloroformic extract produced a reddish-brown interface. Reducing sugars were detected by the 3,5-dinitrosalicylic acid (DNS) method, based on the development of an orange-red color indicating reduction of DNS by aldehyde groups. Finally, anthraquinones were identified by the Bornträger’s test, which yielded a pink to red coloration after treatment with ammonia following chloroform extraction [29,30].

Thin-layer chromatography (TLC) of the lyophilized aqueous extract of *Ipomoea pes-caprae* was performed following the procedure described by Gwatidzo et al. [31], with minor modifications. Five TLC plates (5 × 5 cm; Silica gel 60 F254, Supelco, Bellefonte, PA, USA) were prepared by drawing a baseline 0.5 cm from the lower edge using a soft pencil. Samples of the aqueous extract (1 µL, 50 mg/mL) were applied along this line using a micropipette [31]. For comparative purposes, standard reference compounds, quercetin (Essential Nutrition, Monterrey, Mexico), 4-methylumbelliferone, and anthrone (Sigma-Aldrich, St. Louis, MO, USA), were also spotted at a concentration of 1 mg/mL. Chromatographic development was carried out using either pure chloroform or a chloroform/methanol mixture (CHCl_3_/MeOH, 9:1, *v*/*v*) as the mobile phase within a saturated glass chamber. After solvent migration, the solvent front was immediately marked. The developed plates were visualized under ultraviolet (UV) light at 254 and 365 nm. Subsequently, detection reagents including ceric sulfate, 1% ferric chloride, and 1% ethanolic aluminum chloride were applied to reveal phenolic and flavonoid-type compounds [32,33]. The resulting chromatograms were photographed, and retention factor (Rf) values were calculated as the ratio between the distance traveled by the compound and the solvent front (Rf = compound distance/solvent front distance). Each assay was conducted in triplicate to ensure reproducibility [33].

Spectroscopic analyses were also conducted to further characterize the chemical profile of the lyophilized extract. UV–visible spectra of the aqueous extract (0.1 mg/mL in methanol) were obtained using an Evolution 300 UV–Vis Spectrophotometer (Thermo Fisher Scientific, Madison, WI, USA), employing methanol as the blank. Fourier-transform infrared (FTIR) spectra were recorded using an IRTracer-100 Fourier Transform Infrared Spectrophotometer (Shimadzu Corporation, Kyoto, Japan). In addition, a preliminary proton nuclear magnetic resonance (^1^H NMR) analysis was carried out on a Bruker 400 MHz spectrometer (Bruker BioSpin GmbH, Leipzig, Germany), using deuterated dimethyl sulfoxide (DMSO-d_6_, Sigma-Aldrich, St. Louis, MO, USA) as the solvent. Chemical shifts (δ, ppm) and coupling constants (J, Hz) were reported. The spectral information obtained from the UV, FTIR, and NMR analyses is provided in the Supplementary Material (Figures S1–S4). The analytical techniques employed in this study were intended to provide a preliminary phytochemical characterization of the aqueous infusion and to generate complementary information regarding its major chemical constituents. These analyses were not designed for definitive structural elucidation of individual metabolites.

#### Acid Hydrolysis of IPCAE

Acid hydrolysis of the aqueous lyophilized extract of *Ipomoea pes-caprae* leaves (IPCAE) was performed under reflux conditions. Briefly, the extract was mixed with a 10% (*v*/*v*) hydrochloric acid solution prepared by dilution of concentrated HCl in distilled water and maintained at 60 °C under constant agitation for 72 h. The reaction was carried out in a reflux system to prevent solvent loss during heating. After hydrolysis, the mixture was cooled to room temperature and neutralized as required for subsequent analysis. The hydrolyzed extract was then filtered through Whatman No. 1 filter paper under gravity filtration to remove insoluble material. The filtrate was collected in amber glass containers and stored at 4 °C until further phytochemical and chromatographic evaluation. This hydrolysis procedure was performed exclusively for thin-layer chromatography (TLC) monitoring and comparative phytochemical profiling of the hydrolyzed extract [34,35]. The hydrolysis procedure was intended to promote the cleavage of glycosidic bonds and facilitate the release of aglycone forms of phenolic and flavonoid-related compounds. Comparison of hydrolyzed and non-hydrolyzed extracts was performed to identify potential changes in chromatographic behavior associated with conjugated metabolites. The selected conditions (10% *v*/*v* HCl, 60 °C, 72 h) were chosen to maximize hydrolysis of glycosylated constituents and facilitate comparison between hydrolyzed and non-hydrolyzed extracts. Acid hydrolysis was employed as a phytochemical tool to promote cleavage of glycosidic bonds and release aglycone forms of flavonoid- and phenolic-related compounds for comparative chromatographic evaluation [36,37].

### 2.3. DPPH Radical Scavenging Activity of Polyphenols from IPCAE

The antioxidant activity of the aqueous infusion of *Ipomoea pes-caprae* leaves (IPCAE) was evaluated using the DPPH radical scavenging assay, a widely used method due to its simplicity, rapidity, and reproducibility [33]. The assay was performed following a modified protocol described by Foti, 2015 [38], using ascorbic acid (AC) as the positive control [35]. Sample solutions at different concentrations (0.005–1.0 mg/mL) were mixed with DPPH solution and absolute ethanol. A blank solution containing DPPH and absolute ethanol without sample was used for calibration. The reaction mixtures were incubated in darkness for 30 min at room temperature, and absorbance was measured at 517 nm using a UV-Vis spectrophotometer. Ascorbic acid was used as the positive control, and its radical scavenging activity was considered the reference antioxidant activity (100%) for comparative purposes. Accordingly, the antioxidant activity of the extract was interpreted relative to the activity of the positive control. Radical scavenging activity (%) was calculated as previously described [38,39].

### 2.4. Total Polyphenol Content Determination by the Folin Ciocalteu Method

The total phenolic content (TPC) of the aqueous infusion of *Ipomoea pes-caprae* leaves (IPCAE) was determined using the Folin–Ciocalteu method according to Hudz et al., 2019, and Hernández-Rangel et al., 2024 [40,41]. Briefly, 300 µL of sample were mixed with 1.5 mL of Folin–Ciocalteu reagent (1:10 dilution) and 1.2 mL of 7.5% (*w*/*v*) sodium carbonate. The mixture was incubated for 30 min at room temperature, and absorbance was measured at 765 nm. A blank containing distilled water and Folin–Ciocalteu reagent without sample was used for calibration. Results were expressed as gallic acid equivalents (GAE, mg/100 g of material) using the calibration curve (y = 0.4992x − 0.4812, R^2^ = 0.9885). The IPCAE sample at a concentration of 1.0 mg/mL was analyzed in triplicate [40,41].

### 2.5. Reducing Sugar Content

The reducing sugar content of the aqueous infusion of *Ipomoea pes-caprae* leaves (IPCAE) was determined using the 3,5-dinitrosalicylic acid (DNS) method. A glucose calibration curve was prepared using D-glucose standards (0–500 ppm). Standard solutions were mixed with DNS reagent and distilled water, heated at 80 °C for 10 min, and cooled before absorbance measurement at 540 nm using a UV-Vis spectrophotometer (Thermo Scientific BioMate, Waltham, MA, USA).

For the IPCAE sample, lyophilized infusion material was prepared at 1.0 mg/mL. A 2.0 mL aliquot was mixed with DNS reagent and distilled water, vortexed for 1 min, heated at 80 °C for 10 min, and subsequently cooled for 20 min. A blank solution without D-glucose was used for calibration. All measurements were performed in triplicate using three independent sample preparations to ensure reproducibility. Reducing sugar concentration was calculated from the glucose standard curve and expressed as mg/mL. All reagents were obtained from Sigma-Aldrich [42,43].

### 2.6. Cell Line Model

The MDA-MB-231 human triple-negative breast cancer (TNBC) cell line (ATCC^®^ HTB-26™, Manassas, VA, USA) was used as an in vitro and in vivo tumorigenic model. This cell line lacks expression of estrogen receptor (ER), progesterone receptor (PR), and human epidermal growth factor receptor 2 (HER2), and is widely recognized for its high metastatic potential and use in preclinical TNBC research [44,45].

Cells were cultured in Dulbecco’s Modified Eagle’s Medium (DMEM; Sigma-Aldrich, St. Louis, MO, USA) supplemented with 10% (*v*/*v*) fetal bovine serum (FBS; Gibco, Gaithersburg, MD, USA) and 1× penicillin–streptomycin solution (Antibiotic-Antimycotic 100×; Gibco). Cultures were maintained at 37 °C in a humidified incubator under 5% CO_2_ and 95% air. Subculturing was performed using 0.25% trypsin–EDTA (Gibco), and all manipulations were conducted in a Class II A2 biosafety cabinet to ensure sterility.

### 2.7. Preliminary Acute Oral Toxicity Evaluation

A preliminary acute oral toxicity screening was performed to evaluate the systemic tolerance of the aqueous lyophilized extract of *Ipomoea pes-caprae*. Seven young male BALB/c mice received a single oral administration of the extract at 2000 mg/kg body weight. Animals were continuously monitored during the first 24 h and subsequently observed daily for clinical signs of toxicity, including hypoactivity, piloerection, locomotor impairment, grooming alterations, dermal changes, and mortality [46,47].

No immediate mortality or severe acute toxic manifestations were observed during the initial evaluation period. The experimental dose selected for the subsequent study was based on three complementary considerations. First, it was intended to approximate the traditional preparation and consumption of aqueous infusions of *Ipomoea pes-caprae* used in the study region. Second, a preliminary acute oral toxicity screening performed at 2000 mg/kg did not produce immediate mortality or severe acute toxic manifestations, indicating that lower doses could be evaluated in subsequent experiments. Third, the selected dose falls within the range commonly employed in an exploratory preclinical study of crude botanical extracts. Furthermore, the experimental dose represented only 25% of the screening dose, providing an additional safety margin while maintaining relevance to traditional exposure conditions. The present work was conceived as an exploratory evaluation of biological activity and toxicity rather than a dose-optimization study. Therefore, a lower experimental dose equivalent to 500 mg of dried leaves/kg/day was selected for the TNBC study. This procedure was intended solely as a preliminary toxicity screening and not as a formal LD_50_ determination according to OECD guidelines [46].

### 2.8. In Vivo Tumor Model

Twenty-six female nude (nu/nu) mice, 4 weeks of age, were obtained from the animal facility of the State Cancer Institute of Colima (Colima, Mexico). Animals were housed under specific pathogen-free (SPF) conditions at 21 ± 2 °C, with a 12 h light–dark cycle and ad libitum access to standard food and water. To establish the TNBC tumor model, each mouse received a subcutaneous injection of 1 × 10^7^ MDA-MB-231 cells suspended in 100 µL of sterile phosphate-buffered saline (PBS) into the dorsal flank. Tumor growth was monitored every three days until tumors reached a diameter of 4–6 mm, at which point treatment was initiated [48,49].

### 2.9. Experimental Design and Treatments

The study was designed as a prospective, randomized preclinical trial following the ARRIVE Essential 10 guidelines [44]. When tumors reached 4–6 mm in diameter, mice were randomly allocated into three treatment groups: Control group (*n* = 8): received 100 µL of sterile saline solution orally once daily for 30 days.

IPCAE group (*n* = 10): received a freshly prepared aqueous infusion of *Ipomoea pes-caprae* leaves equivalent to 500 mg of dried leaves/kg/day, administered orally in a volume of 100 µL. Based on the extraction yield obtained after lyophilization, this dose corresponded to approximately 56 mg/kg/day of lyophilized extract. Treatment was originally planned to continue throughout the entire 30-day experimental period; however, administration was discontinued after 15 consecutive days because of severe treatment-related toxicity, including extensive dermal lesions, marked clinical deterioration, and increased mortality. Continuation of treatment beyond this point was considered incompatible with animal welfare considerations and humane endpoint criteria.

Cisplatin group (*n* = 8): received cisplatin (5 mg/kg, intraperitoneally) on days 1, 8, 15, and 22 [48,49]. This dosing schedule was selected according to previously published xenograft protocols employing intermittent administration to maintain antitumoral activity while limiting cumulative toxicity [6,50,51].

Tumor growth was monitored throughout the experimental period or until humane endpoint criteria were reached, including tumor diameter ≥ 25 mm, severe clinical deterioration, or marked reduction in mobility and activity [51].

### 2.10. Outcome Measures

Tumor volume was measured every five days using digital calipers and calculated according to the formula: V = (π/6)*D*d^2^; where D is the major tumor diameter and d is the minor diameter. Survival was recorded daily and analyzed using Kaplan–Meier survival curves. Adverse clinical signs (e.g., skin lesions with fluid accumulation, adynamia, or reduced activity) were monitored daily. Animals that reached endpoint criteria were humanely euthanized using pentobarbital anesthesia (ARANDA, Jalisco, Mexico), followed by cervical dislocation, in accordance with the AVMA Guidelines for the Euthanasia of Animals (2013) [52].

### 2.11. Statistical Analysis

Statistical analysis was performed using SPSS version 22 software (IBM Corp; Armonk, NY, USA). The Shapiro–Wilk test was applied to determine the distribution of the data [53]. To assess differences between treatments in terms of tumor growth, the Kruskal–Wallis test was used, followed by the Mann–Whitney U test as a post hoc test. A statistically significant difference was considered when the *p* value was less than 0.05. Survival was analyzed using the Kaplan–Meier method, supplemented with log-rank tests to contrast survival curves between groups. Results are presented as median and range or 25–75th percentile (Q1–Q3) or 95% CI to describe the central tendency and dispersion of the data, respectively [54].

### 2.12. Ethical Statement

The animal study protocol was approved by the Comité de Ética en Investigación and the Comité Interno para el Cuidado y Uso de los Animales de Laboratorio (CICUAL) of the Instituto Estatal de Cancerología del Estado de Colima (protocol code: CICANCL080520-IPOMPE5; date of approval: 8 May 2020). The study was conducted in accordance with the Guide for the Care and Use of Laboratory Animals (National Academy of Sciences, USA, 2011) and complied with the Official Mexican Standard for the Use and Care of Laboratory Animals (NOM-062-ZOO-1999) [55].

## 3. Results

### 3.1. Phytochemical Characterization and Spectroscopic Profile of the Aqueous Infusion of IPCAE

From 100 mL of aqueous infusion prepared from dried and pulverized *Ipomoea pes-caprae* leaves, a total of 56 mg of reddish-brown lyophilized semisolid extract was obtained after infusion preparation, lyophilization, and solvent evaporation, corresponding to a yield of 11.2% (*w*/*w*). The extract exhibited a dark green to reddish-brown coloration and a semisolid consistency.

The qualitative phytochemical analysis of the IPCAE leaves revealed the presence of several major classes of secondary metabolites (Table 1). Strong reactions were observed for flavonoids and reducing sugars, indicating their abundance in the extract. The flavonoid content was evidenced by an intense color change in both the Shinoda and Marini Bettolo tests, suggesting the presence of flavonols or flavones [56,57]. Reducing sugars, quantified using the DNS method, showed a concentration of 124.56 ± 4.5 µg/mL (glucose equivalents), confirming a high level of carbohydrate derivatives with reducing potential.

Moderate reactions were obtained for saponins, characterized by stable foam formation and partial hemolysis in the blood agar assay, while weak reactions were detected for tannins and terpenoids, as indicated by a faint bluish-black coloration with ferric chloride and a reddish-brown interface in the Salkowski test, respectively. In contrast, alkaloids and anthraquinones were not detected under the tested conditions, suggesting that these metabolite classes are either absent or present in negligible amounts in lyophilized infusion.

The FTIR spectrum shows several characteristic absorption bands corresponding to key functional groups. A broad signal around 3306 cm^−1^ indicates the presence of O–H stretching vibrations, suggesting hydroxyl groups from alcohols or phenols involved in hydrogen bonding. The peaks at 2921 and 2852 cm^−1^ correspond to C–H stretching of aliphatic chains, typical of methyl and methylene groups. A distinct and strong absorption at 1733 cm^−1^ confirms the presence of a carbonyl (C=O) group, which is characteristic of esters, aldehydes, or ketones. Additional bands at 1605 and 1517 cm^−1^ are associated with C=C stretching of aromatic rings, while signals at 1449 and 1376 cm^−1^ correspond to C–H bending vibrations. The region between 1237 and 1070 cm^−1^ shows intense peaks attributed to C–O and C–O–C stretching, typical of alcohols, ethers, or ester linkages. Finally, the signals below 900 cm^−1^ correspond to aromatic C–H out-of-plane bending, confirming the presence of substituted aromatic structures. Overall, the spectrum suggests a complex organic compound containing hydroxyl, carbonyl, aromatic, and ether groups, consistent with a polyphenolic or ester-type structure [31,58,59,60].

The preliminary ^1^H-NMR spectrum of the crude IPCAE leaves showed characteristic signals suggesting the presence of multiple classes of secondary metabolites, including glycosidic and polyphenolic compounds. The resonances observed between 3.0 and 5.5 ppm correspond to protons attached to oxygenated carbons, typical of carbohydrate or glycosidic structures, while signals in the aromatic region (6.5–7.8 ppm) indicate the presence of flavonoid-like aromatic protons. Additionally, intense aliphatic signals between 0.8 and 2.0 ppm suggest the coexistence of lipidic components. These findings support the qualitative phytochemical results, although further structural elucidation by 2D-NMR and mass spectrometry (MS) analyses is required to confirm the identity of compounds such as cairicoside I, batatins, or related polyphenolic glycosides [59].

### 3.2. Polyphenol Content, Antioxidant Activity, and Reducing Sugar Determination of IPCAE

The aqueous infusion of *Ipomoea pes-caprae* leaves was obtained as a brown lyophilized powder and standardized at 1 mg/mL for analysis. The infusion showed a total polyphenol content of 7.29 µg GAE/mg extract, equivalent to 7.29 µg GAE/mL of the evaluated infusion, and demonstrated notable antioxidant activity with 72.25 ± 1.25% DPPH radical inhibition. Additionally, the DNS assay revealed a reducing sugar concentration of 124.56 ± 4.5 µg/mL, evidenced by a strong orange-red coloration Table 2.

### 3.3. Chromatographic Analysis

The TLC (Figure S5) profile revealed notable differences between the non-hydrolyzed extract (IPCAE) and the hydrolyzed extract (IPCAEh). Under UV light at 365 nm (plate A), IPCAEh displayed three major fluorescent spots with approximate Rf values of 0.60, 0.44, and 0.26, whereas IPCAE showed a less defined chromatographic profile with a predominant signal near Rf 0.88. Under UV light at 254 nm (plate B), additional absorption bands were observed in IPCAEh, particularly at Rf values of approximately 0.78 and 0.40, indicating the presence of aromatic or conjugated structures. After derivatization with aluminum chloride (plate C), a faint yellow fluorescent band was observed at low retention (Rf ≈ 0.10), supporting the presence of flavonoid-related compounds. Dragendorff’s reagent (plate D) produced only weak basal staining, suggesting the absence or low abundance of alkaloids [61].

**Figure 1:**
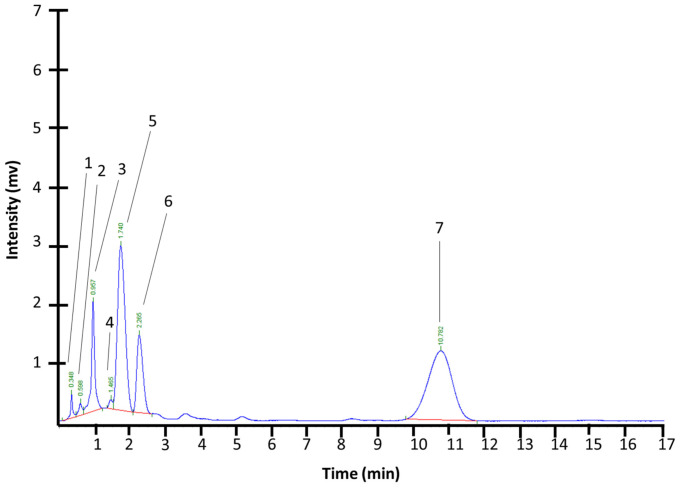
HPLC chromatographic profile of the aqueous extract of *Ipomoea pes-caprae* leaves (IPCAE) detected at 290 nm using a Symmetry C18 column (5 µm, 4.6 × 250 mm). The mobile phase consisted of acetonitrile:methanol:acidified water containing 0.5% phosphoric acid (25:25:50, *v*/*v*/*v*). The blue trace corresponds to the chromatographic profile of the IPCAE sample, whereas the red trace represents the chromatographic baseline generated by the instrument software. Peaks 1–7 correspond to the major chromatographic signals detected in the extract. The predominant peaks were observed at retention times (TR) of 1.740 and 10.782 min. Retention times and peak areas (AUC) are summarized in Table 3. The chromatographic profile is presented for comparative phytochemical characterization and does not represent definitive identification of individual compounds.

HPLC analysis of IPCAE revealed seven major chromatographic peaks, with predominant signals detected at retention times of 1.740 min and 10.782 min, corresponding to relative areas of 31.98% and 42.99%, respectively (Table 3, Figure 1). Comparative analysis with flavonoid-related standards suggested the presence of structurally related phenolic compounds; however, definitive metabolite identification was not possible under the present experimental conditions and requires further confirmation by LC-MS/MS and co-injection analyses with purified standards [32,62,63].

### 3.4. Preliminary Acute Toxicity Evaluation, Tumor Progression, Adverse Effects, and Survival

Preliminary acute toxicity screening of the aqueous lyophilized extract of *Ipomoea pes-caprae* showed that oral administration at 2000 mg/kg did not produce immediate mortality or severe acute toxic manifestations in BALB/c mice during the initial observation period. Based on these findings, a lower experimental dose equivalent to 500 mg of dried leaves/kg/day was selected for the TNBC study to approximate traditional ethnomedicinal exposure conditions. Considering the extraction yield obtained from the aqueous infusion, where 56 mg of reddish-brown lyophilized semisolid extract were recovered from 500 mg of dried leaves (11.2% *w*/*w*), the administered dose corresponded approximately to 56 mg/kg/day of lyophilized extract.

Based on the ethnomedical use of *I. pes-caprae* in the Tecomán, Colima region, where leaf infusions are traditionally employed for kidney pain, inflammatory conditions, rheumatism, and occasionally for venomous bites or tumor-related ailments, the experimental preparation was designed to resemble the traditional aqueous infusion used by local populations. Dried leaves were ground and prepared as an aqueous infusion to approximate the traditional route of administration and exposure conditions.

**Figure 2:**
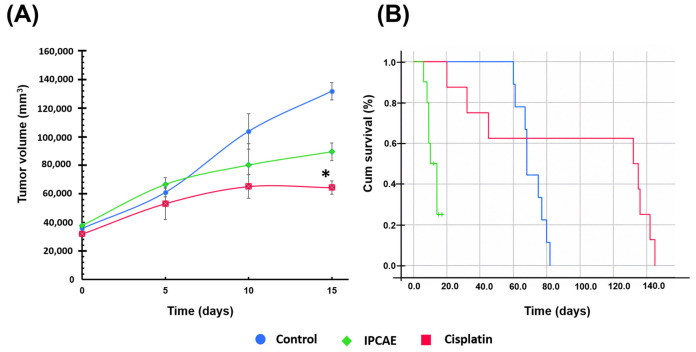
Tumor progression and survival in mice bearing MDA-MB-231 xenografts. (**A**) Tumor volume progression in mice treated with saline control, IPCAE, or cisplatin. Data are presented as mean ± SD. * *p* < 0.05 versus Control group. Tumor volume comparisons were performed using the Kruskal–Wallis test followed by Mann–Whitney U post hoc tests. (**B**) Kaplan–Meier survival curves for the same treatment groups. Survival distributions were compared using the log-rank test. No statistically significant differences in tumor volume were observed between the IPCAE and control groups during the study period. Survival was significantly reduced in the IPCAE group compared with both the control and cisplatin groups (*p* < 0.001).

At day 0, no significant differences in tumor volume were observed among groups (*p* > 0.05; Figure 2). By day 5, cisplatin-treated mice exhibited significantly smaller tumor volumes compared with the IPCAE group (*p* = 0.003), whereas no significant differences were detected between the control and IPCAE groups (*p* = 0.295). At day 10, no statistically significant differences in tumor volume were observed among groups (*p* > 0.05). By day 15, cisplatin significantly reduced tumor volume compared with the control group (*p* = 0.004), while IPCAE did not produce a statistically significant reduction compared with either the control group (*p* = 0.214) or the cisplatin group (*p* = 0.377). Although a slight trend toward slower tumor progression was observed in the IPCAE-treated animals during the first treatment days, this effect was transient and accompanied by marked systemic toxicity after day 15.

Importantly, mice treated with IPCAE developed marked systemic toxicity. Approximately 40% (4/10) of animals developed severe ulcerative and blister-like thoracic dermal lesions by day 10, accompanied by hypoactivity and progressive clinical deterioration. By day 15, mortality reached 70% in the IPCAE-treated group. No comparable lesions or mortality were observed in control or cisplatin-treated animals.

Kaplan–Meier survival analysis confirmed significantly lower survival in the IPCAE group compared with both control and cisplatin groups (*p* < 0.001). Although a transient numerical tendency toward reduced tumor progression was observed during the first treatment days, repeated administration of IPCAE resulted in marked systemic toxicity without demonstrating statistically significant antitumoral activity under the evaluated experimental conditions.

## 4. Discussion

In this study, the findings are relevant for the growing use of medicinal plant-based functional preparations and nutraceutical products. The phytochemical characterization of the aqueous infusion of *Ipomoea pes-caprae* leaves (IPCAE) revealed a complex composition enriched in flavonoids, reducing sugars, and saponins, compounds commonly associated with antioxidant, anti-inflammatory, and cytotoxic activities. The strong positive reactions observed in the Shinoda and Marini-Bettolo assays suggest the predominance of flavonols and flavones previously reported in *I. pes-caprae* and other medicinal plants traditionally used in coastal and tropical regions. These findings are consistent with the antioxidant activity observed in the DPPH assay, where the infusion showed 72.25 ± 1.25% radical inhibition, likely associated with the presence of phenolic hydroxyl groups capable of donating electrons and neutralizing reactive oxygen species. Considering that ascorbic acid was used as the reference antioxidant and assigned a relative activity of 100%, the observed 72.25% inhibition indicates moderate to high radical scavenging capacity for crude aqueous plant preparation. This result supports the presence of biologically relevant antioxidant constituents and is consistent with the flavonoids and phenolic compounds detected during phytochemical characterization [64,65].

The FTIR and preliminary ^1^H-NMR analyses further support the presence of polyphenolic glycosides and carbohydrate-derived metabolites. Characteristic O–H stretching vibrations, aromatic C=C signals, and glycosidic proton resonances suggest the presence of flavonoid-related, phenolic, and glycosylated constituents. However, the chromatographic and spectroscopic analyses performed in this study do not allow definitive identification of individual metabolites. Therefore, references to compounds previously reported in *I. pes-caprae*, including cairicosides, batatins, and related glycosylated metabolites, should be considered only as examples of constituents described in the literature and not as compounds identified in the present extract [66,67]. These approaches were intended as exploratory characterization tools rather than definitive structural elucidation methods. Therefore, further LC-MS/MS and 2D-NMR studies are required to confirm the identity of the metabolites present in the extract [66,68,69,70].

An important aspect of this study is that *I. pes-caprae* is commonly used in traditional medicine for multiple ailments, including inflammatory disorders, kidney pain, rheumatic conditions, skin lesions, and insect stings. The present findings indicate that biological responses may differ when preparations are standardized and repeatedly administered under experimental conditions [71,72].

Although a transient numerical tendency toward slower tumor progression was observed during the first treatment days, repeated oral administration of IPCAE at 500 mg of dried leaves/kg/day produced marked systemic toxicity, including progressive clinical deterioration, severe dermal lesions, and elevated mortality in tumor-bearing mice. These findings should be interpreted cautiously because the physiological condition of tumor-bearing immunodeficient animals may influence susceptibility to adverse effects [73,74,75]. Cancer-associated metabolic alterations, chronic inflammation, immunological impairment, and the physiological burden imposed by tumor progression may modify the toxicological response compared with healthy animals or traditional users. Therefore, the toxicity observed under the present experimental conditions should not be directly extrapolated to traditional human use, which typically involves different preparation methods, lower concentrations, less frequent administration, and distinct exposure patterns [68,75].

Nevertheless, these findings reinforce the importance of systematically evaluating the safety of medicinal plants under standardized experimental conditions, as traditional use alone should not be interpreted as evidence of safety across different doses, formulations, or therapeutic settings.

The pharmacological behavior of medicinal plant preparations depends on multiple variables, including the species, plant part used, extraction method, preparation conditions, route of administration, exposure duration, and administered dose. Traditional preparations often involve low-concentration infusions consumed intermittently, whereas experimental preparations may expose organisms to higher and more standardized concentrations of bioactive metabolites. Consequently, the severe toxicity observed in the present study highlights the importance of conducting rigorous toxicological evaluations before considering systemic therapeutic applications of this preparation [73,76,77].

The coexistence of antioxidant activity and systemic toxicity may be explained by the chemical complexity of the crude extract. While flavonoids and other phenolic constituents have been associated with antioxidant and cytoprotective activities in previous studies, the specific metabolites responsible for the biological effects observed in the present study remain unknown because definitive compound identification was beyond the scope of this work [67,68,69]. Importantly, no statistically significant antitumoral effect was demonstrated under the experimental conditions evaluated. Therefore, any apparent differences in tumor progression should be interpreted as exploratory observations rather than evidence of therapeutic efficacy. The observed combination of antioxidant activity and systemic toxicity underscores the need for fractionation and purification strategies aimed at identifying safer and better-characterized constituents.

An important limitation of the present study is that the preliminary toxicity assessment was not designed as a formal OECD-compliant acute toxicity study and therefore did not allow comprehensive characterization of the toxicological profile of the extract before initiation of the antitumoral experiment. Consequently, the selected dose should be considered exploratory in nature, and its safety profile cannot be regarded as fully established prior to treatment. Unexpectedly, repeated administration of the traditionally prepared infusion produced marked systemic toxicity, including progressive clinical deterioration characterized by piloerection, lacrimation, hypoactivity, social isolation, occasional aggressive behavior, severe dermal lesions, and ultimately high mortality. The severity of these adverse effects substantially reduced the number of surviving animals, preventing the generation of sufficiently robust data for meaningful histopathological, biochemical, or mechanistic analyses. Therefore, although such evaluations would have strengthened the biological interpretation of the observed toxicity, any results obtained under these circumstances would have lacked adequate statistical power and could have led to unreliable conclusions. Future investigations should prioritize comprehensive toxicological characterization following internationally accepted guidelines, including dose-escalation studies, repeated-dose toxicity, serum biochemical markers, histopathological evaluation, pharmacokinetic analyses, and bioassay-guided phytochemical fractionation to identify the metabolites responsible for the observed biological effects. Importantly, the principal contribution of the present study is not to challenge the traditional use of *Ipomoea pes-caprae*, but rather to demonstrate that traditional use alone should not be considered sufficient evidence of safety under standardized experimental conditions or repeated systemic administration. Reporting these unexpected findings contributes to reducing publication bias and provides valuable information for the rational and safe evaluation of medicinal plants intended for future therapeutic or nutraceutical applications.

Collectively, these findings provide additional information regarding the phytochemical characteristics, antioxidant activity, and toxicity profile of *Ipomoea pes-caprae*. Although the present work was originally conceived to explore the potential antitumoral activity of a traditionally consumed preparation, the unexpected systemic toxicity observed under the evaluated experimental conditions shifted the principal biological significance of the study toward safety considerations. Rather than contradicting its long-standing ethnomedicinal use, these findings indicate that traditional use alone should not be considered sufficient evidence of safety under standardized experimental conditions or repeated systemic administration. Future studies should prioritize comprehensive toxicological characterization, including dose-escalation studies, repeated-dose toxicity, histopathological evaluation, serum biochemical analyses, pharmacokinetic assessment, and bioassay-guided phytochemical fractionation to identify the metabolites responsible for the observed biological effects. In addition, evaluating lower doses and comparing traditional patterns of exposure with controlled experimental regimens may help clarify the relationship between preparation, dosage, host physiological status, and toxicity. Reporting these findings contributes to the rational and safe scientific evaluation of medicinal plants and highlights the importance of publishing unexpected toxicological outcomes alongside studies of potential therapeutic efficacy.

## 5. Conclusions

The aqueous infusion of *Ipomoea pes-caprae* leaves (IPCAE) exhibited a phytochemical profile characterized by abundant flavonoids and reducing sugars, together with notable antioxidant activity. However, under the experimental conditions evaluated, no statistically significant antitumoral activity was demonstrated in the TNBC xenograft model, whereas repeated oral administration produced marked systemic toxicity characterized by progressive clinical deterioration and high mortality. These findings further indicate that traditional medicinal use alone should not be considered sufficient evidence of safety under standardized experimental conditions or repeated systemic administration. Although the present study was originally designed to explore the potential antitumoral activity of a traditionally consumed preparation, the observed toxicity highlights the importance of systematically evaluating both efficacy and safety during the preclinical development of medicinal plants. Future studies should include comprehensive toxicological characterization, dose-escalation and dose-optimization studies, histopathological and biochemical evaluation, pharmacokinetic analyses, and bioassay-guided fractionation to identify the metabolites responsible for the observed biological effects before considering potential therapeutic or nutraceutical applications of *I. pes-caprae*.

## Figures and Tables

**Table 1:** Summary of the phytochemical analysis in the infusion extract of *Ipomoea pes-caprae* leaves (IPCAE).

Metabolites	IPCAE (1 mg/mL)	Observation/Reaction Intensity
Alkaloids	–	Not detected with Dragendorff’s, Mayer’s, and Wagner’s reagents
Flavonoids	+++	Strong positive reaction (Shinoda and Marini Bettolo tests)
Saponins	++	Moderate foam formation and partial hemolysis on blood agar
Tannins	+	Weak bluish-black coloration with FeCl_3_
Terpenoids	+	Weak reddish-brown interface in Salkowski test
Anthraquinones	–	Not detected by Bornträger’s test

All experiments were performed in triplicate. Symbol key: strong intensity reaction (+++), medium intensity reaction (++), weak intensity reaction (+), not detected (–). Quantitative result for reducing sugars is expressed as glucose equivalents (µg/mL, mean ± SD, *n* = 3).

**Table 2:** Phytochemical characterization, antioxidant activity, and reducing sugar content of IPCAE.

Test	Concentration/Result	Reaction/Observation
Total polyphenol content (Folin–Ciocalteu method)	7.29 µg GAE/mg extract (equivalent to 7.29 µg GAE/mL infusion)	Positive reaction for phenolic compounds; the infusion appeared as a brown powder after lyophilization
Antioxidant activity (DPPH assay)	72.25 ± 1.25% radical inhibition at 1 mg/mL extract concentration	Strong antioxidant capacity with notable DPPH radical scavenging activity
Reducing sugars (DNS method)	124.56 ± 4.5 µg/mL	Strong orange-red coloration observed at 1 mg/mL extract concentration

Values are expressed as mean ± standard deviation of triplicate analyses. GAE: gallic acid equivalents; DPPH: 2,2-diphenyl-1-picrylhydrazyl; DNS: 3,5-dinitrosalicylic acid. Antioxidant activity was evaluated at an extract concentration of 1 mg/mL.

**Table 3:** HPLC chromatographic profile of the aqueous extract of *Ipomoea pes-caprae* leaves (IPCAE).

Peak No.	Retention Time (min)	Peak Height	Peak Area	Relative Area (%)
1	0.348	383.730	1597.150	1.2249
2	0.598	197.212	1363.244	1.0455
3	0.957	1859.102	12,843.156	9.8495
4	1.465	145.250	1046.369	0.8025
5	1.740	2801.125	41,696.031	31.9771
6	2.265	1315.563	15,794.600	12.1130
7	10.782	1171.277	56,052.898	42.9875
Total		7873.258	130,393.448	100.0000

Chromatographic separation was performed using a Symmetry C18 column (5 µm, 4.6 × 250 mm) with detection at 290 nm. The mobile phase consisted of acetonitrile:methanol:acidified water (25:25:50, *v*/*v*/*v*) containing 0.5% phosphoric acid. Relative area (%) corresponds to the normalized peak area percentage of each detected compound.

## Data Availability

The original contributions presented in the study are included in the article/Supplementary Material; further inquiries can be directed to the corresponding authors.

## Supplementary Materials

The following supporting information can be downloaded at https://www.mdpi.com/article/10.3390/nu18142248/s1. Figure S1. UV–Vis spectrum of the aqueous lyophilized extract of *Ipomoea pes-caprae* leaves (IPCAE) at 0.12 mg/mL. Figure S2. FTIR of the aqueous lyophilized of IPCAE. Figure S3. ^1^H-NMR spectrum (400 MHz) of IPCAE at 0.12 mg/mL in dimethyl sulfoxide-d6 (DMSO-d6). Figure S4. NMR ^1^H spectrum at 400 MHz of IPCAE at 0.12 mg/mL in dimethyl sulfoxide-d6 (expansion 0 to 7.5 ppm). Figure S5. Thin-layer chromatography (TLC) profile of IPCAE and IPCAEh.
