# Supplementary material for: Antioxidant Activity and Dose-Dependent Toxicity of a Traditionally Consumed Ipomoea pes-caprae Infusion Evaluated in a Triple-Negative Breast Cancer Xenograft Model
Source: Nutrients. 2026 Jul 9;18(14):2248. doi: 10.3390/nu18142248 (PMC13415641; doi:10.3390/nu18142248)
Supplement: Supplementary file 1 [file nutrients-18-02248-s001.zip › nutrients-4363038-supplementary.pdf]

Supplementary material

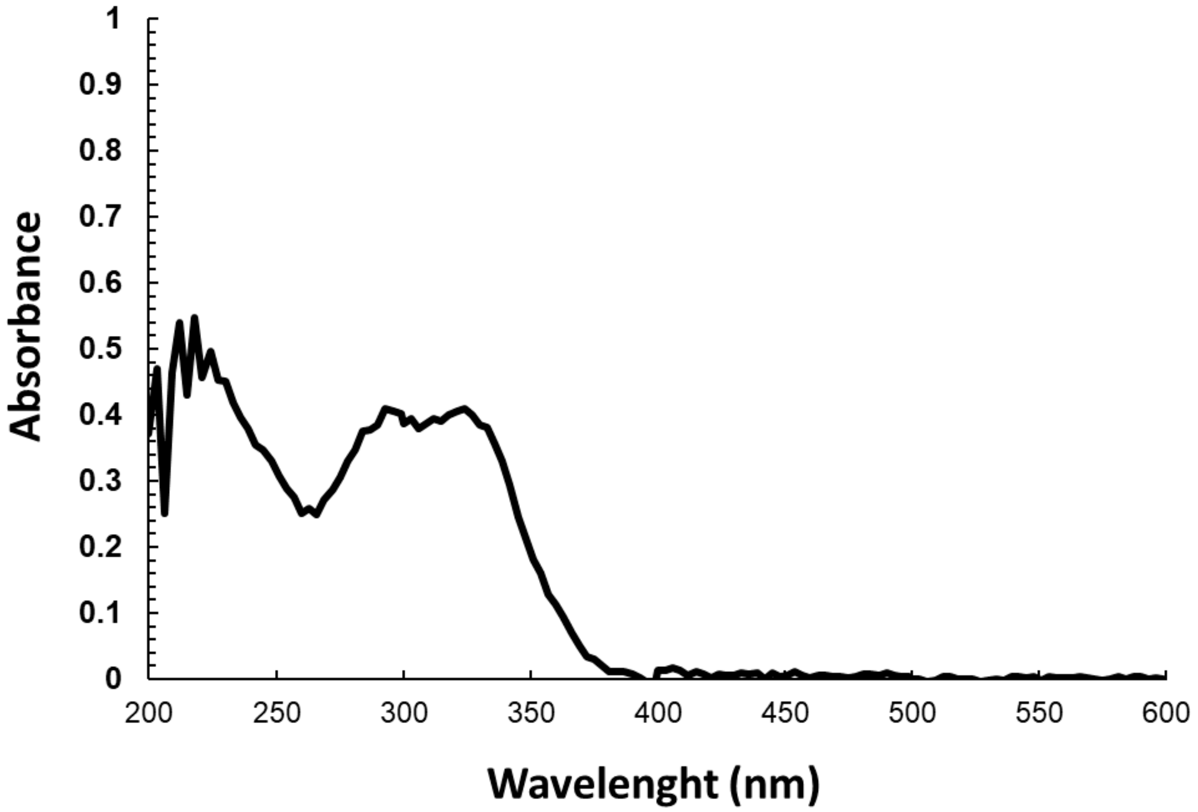

Figure S1. UV–Vis spectrum of the aqueous lyophilized *Ipomoea pes-caprae* at 0.12 mg/mL.

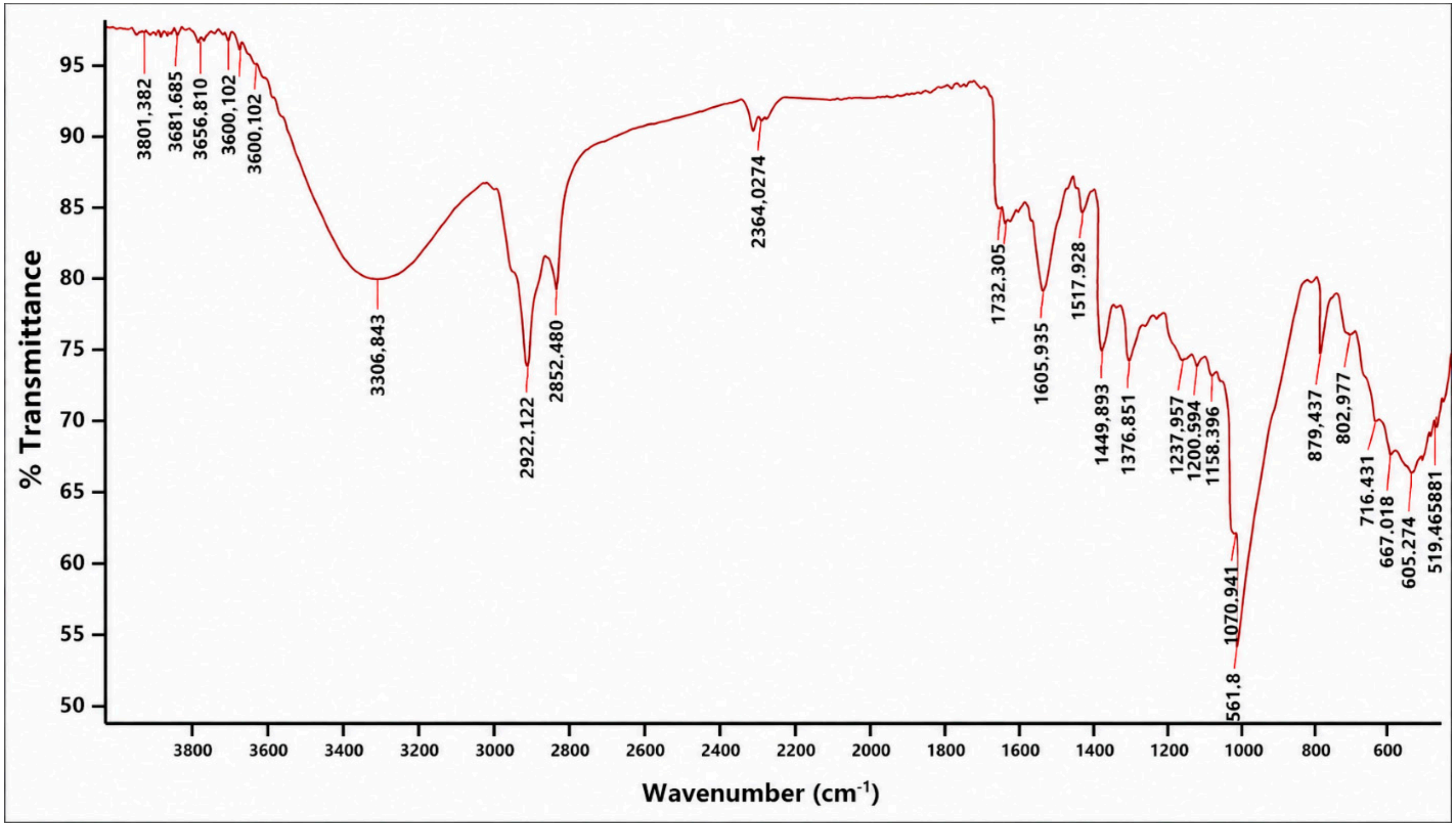

Figure S2. FTIR of the aqueous lyophilized of IPCAE

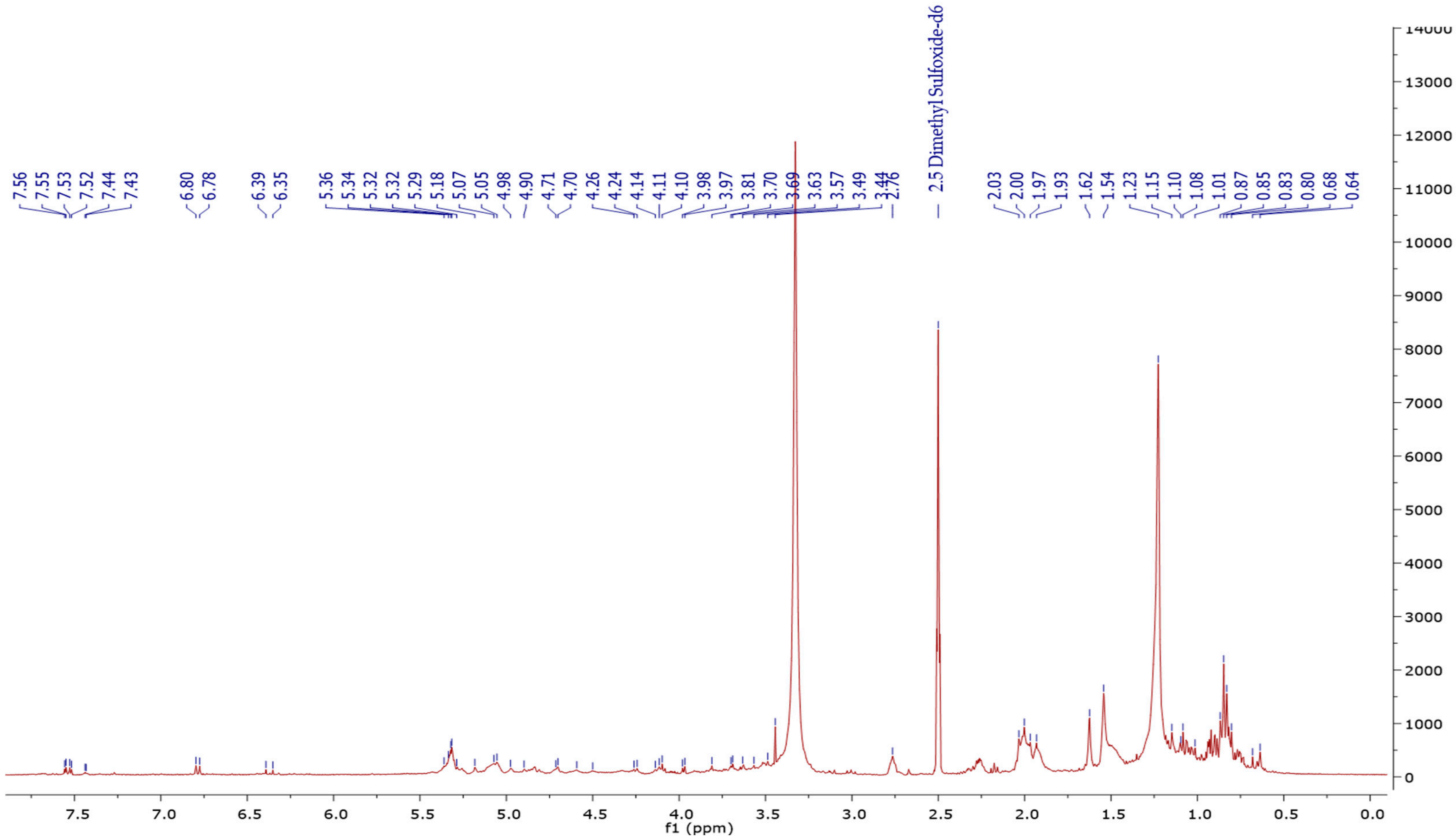

Figure S3. ^{1}H-NMR spectrum (400 MHz) of IPCAE at 0.12 mg/mL in dimethyl sulfoxide-d6 (DMSO-d6).

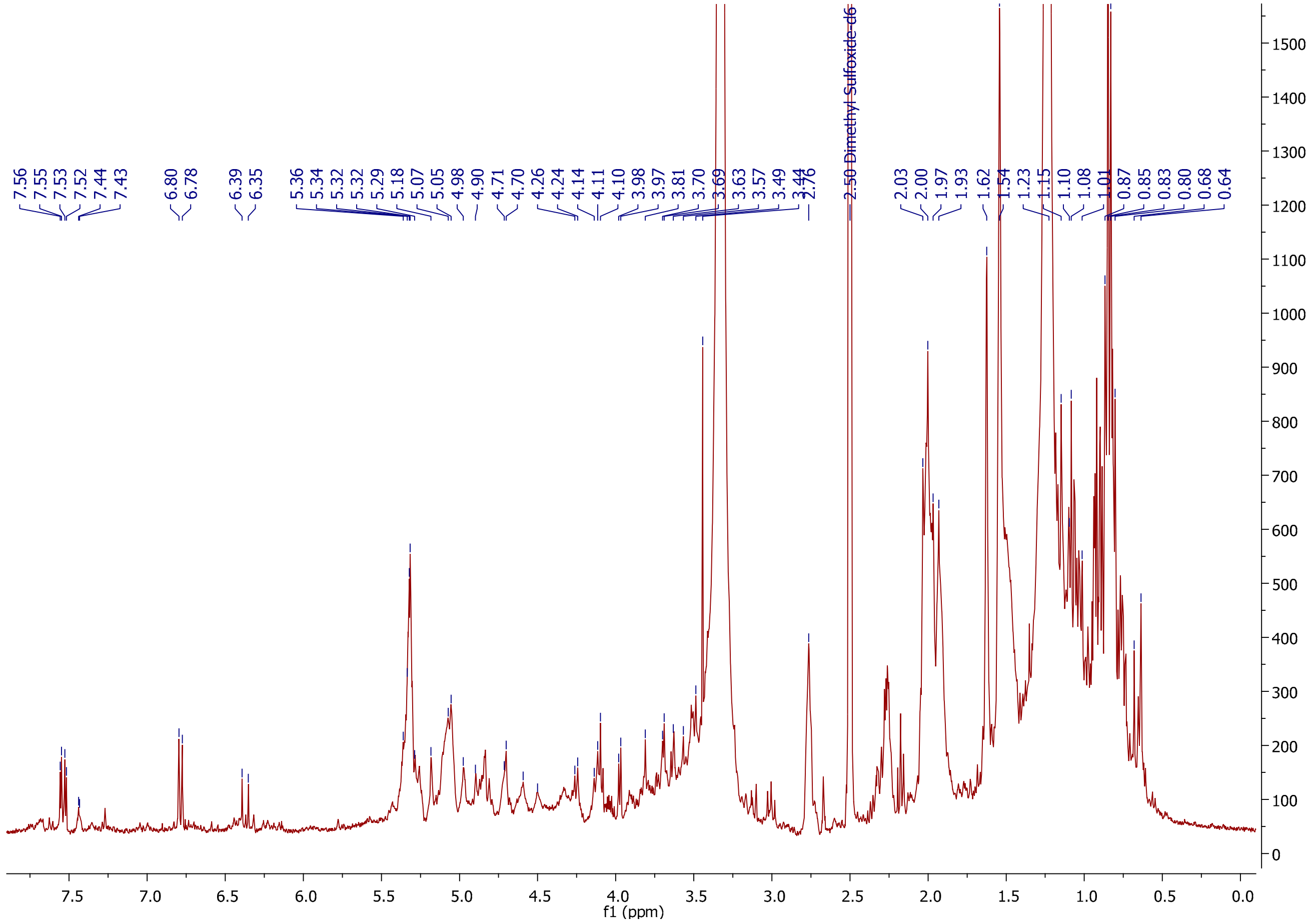

Figure S4. NMR ^{1}H spectrum at 400 MHz of IPCAE at 0.12 mg/mL in dimethyl sulfoxide-d6 (expansion 0 to 7.5 ppm).

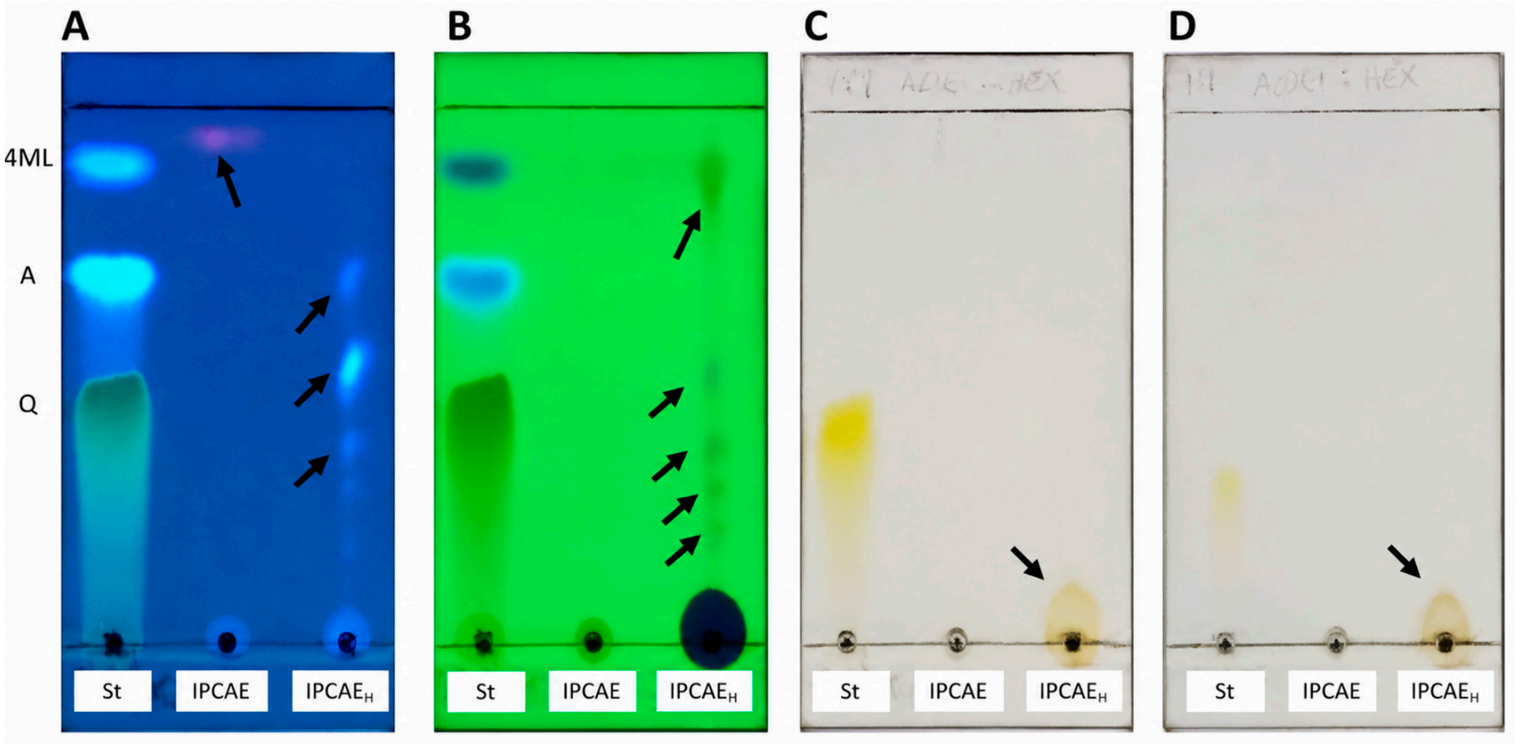

Fig S5. Thin-layer chromatography (TLC) profile of IPCAE and IPCAEh. The samples were eluted using a hexane:ethyl acetate (1:1, v/v) mobile phase. As reference standards, quercetin (Q), anthrone (Ant), and 4-methylumbelliferone (4ML) were used at a concentration of 1 mg/mL. The plates were visualized under (A) long-wave UV light (365 nm), (B) short-wave UV light (254 nm), and after derivatization with (C) aluminum chloride and (D) Dragendorff's reagent. Arrows indicate the spots observed under each visualization condition.
